# 1,25-dihydroxyvitamin-D_3_ distinctly impacts the paracrine and cell-to-cell contact interactions between hPDL-MSCs and CD4^+^ T lymphocytes

**DOI:** 10.3389/fimmu.2024.1448597

**Published:** 2024-09-20

**Authors:** Christian Behm, Oliwia Miłek, Katharina Schwarz, Xiaohui Rausch-Fan, Andreas Moritz, Oleh Andrukhov

**Affiliations:** ^1^ Competence Center for Periodontal Research, University Clinic of Dentistry, Medical University of Vienna, Vienna, Austria; ^2^ Clinical Division of Conservative Dentistry and Periodontology, University Clinic of Dentistry, Medical University of Vienna, Vienna, Austria

**Keywords:** mesenchymal stromal cells, periodontal ligament, immunomodulation, 1,25-dihydroxyvitamin-D_3_, paracrine mechanisms, direct cell-to-cell contact, pro-inflammatory cytokines

## Abstract

**Introduction:**

Human periodontal ligament-derived mesenchymal stromal cells (hPDL-MSCs) possess a strong ability to modulate the immune response, executed via cytokine-boosted paracrine and direct cell-to-cell contact mechanisms. This reciprocal interaction between immune cells and hPDL-MSCs is influenced by 1,25-dihydroxyvitamin-D_3_ (1,25(OH)_2_D_3_). In this study, the participation of different immunomodulatory mechanisms on the hPDL-MSCs-based effects of 1,25(OH)_2_D_3_ on CD4^+^ T lymphocytes will be elucidated using different co-culture models with various cytokine milieus.

**Material and methods:**

hPDL-MSCs and CD4^+^ T lymphocytes were co-cultured indirectly and directly with inserts (paracrine interaction only) or directly without inserts (paracrine and direct cell-to-cell contact interaction). They were stimulated with TNF-α or IL-1β in the absence/presence of 1,25(OH)_2_D_3_. After five days of co-cultivation, the CD4^+^ T lymphocyte proliferation, viability, and cytokine secretion were analyzed. Additionally, the gene expression of soluble and membrane-bound immunomediators was determined in hPDL-MSCs.

**Results:**

In the indirect and direct co-culture model with inserts, 1,25(OH)_2_D_3_ decreased CD4^+^ T lymphocyte proliferation and viability. The direct co-culture model without inserts caused the opposite effect. 1,25(OH)_2_D_3_ mainly decreased the CD4^+^ T lymphocyte-associated secretion of cytokines via hPDL-MSCs. The degree of these inhibitions varied between the different co-culture setups. 1,25(OH)_2_D_3_ predominantly decreased the expression of the soluble and membrane-bound immunomediators in hPDL-MSCs to a different extent, depending on the co-culture models. The degree of all these effects depended on the absence and presence of exogenous TNF-α and IL-1β.

**Conclusion:**

These data assume that 1,25(OH)_2_D_3_ differently affects CD4^+^ T lymphocytes via the paracrine and direct cell-to-cell contact mechanisms of hPDL-MSCs, showing anti- or pro-inflammatory effects depending on the co-culture model type. The local cytokine microenvironment seems to be involved in fine-tuning these effects. Future studies should consider this double-edged observation by executing different co-culture models in parallel.

## 1 Introduction

Mesenchymal stromal cells (MSCs) are multipotent, non-hematopoietic progenitor cells showing a fibroblast-like morphology and self-renewal potential (1). Following the International Society for Cell and Gene Therapy (ISCT), these cells are plastic adherent, have an osteogenic, adipogenic, and chondrogenic differentiation potential *in vitro*, express a specific surface marker set, including CD29, CD73, CD90, CD105, and CD146, and do not express hematopoietic markers, including CD14, CD31, CD34, and CD45 (2, 3). They reside in various human tissues, including bone marrow, adipose tissue, and umbilical cord (4). Additionally, MSCs can also be isolated from multiple dental tissues, like the periodontal ligament (PDL) (5), a highly specialized connective tissue linking the cementum with the alveolar bone (6). Like MSCs from other tissues, human periodontal ligament-derived MSCs (hPDL-MSCs) meet the ISCT-based criteria for MSCs. They are also in a quiescent, undifferentiated state, residing in the perivascular niche of the PDL (7, 8). Since hPDL-MSCs derive from the neural crest, they can also differentiate into neuron-like cells (9). Inflammatory processes within the periodontal tissues shift hPDL-MSCs into an active state, initiating the migration to the injured tissue site through chemotactic processes. There, they control periodontal tissue homeostasis, inflammation, and regeneration, mainly by affecting local innate and adaptive immune responses comparable to MSCs’ functions in other tissues (10, 11).

These mainly immunosuppressive immunomodulatory activities are primarily executed by paracrine factors or direct cell-to-cell contacts. The paracrine mechanisms consist of the secretion of soluble immunomediators and enzymes, including indoleamine-2,3-dioxygenase-1 (IDO-1), prostaglandin E2 (PGE_2_), and tumor necrosis factor-inducible gene 6 protein (TSG-6). The direct cell-to-cell contacts are regulated by membrane-anchored proteins, including programmed cell death 1 ligand 1 (PD-L1) and programmed cell death 1 ligand 2 (PD-L2), which are encoded by the CD274 and CD273 genes, respectively (4, 12). Under homeostatic conditions, the expression of these immunomediators is low but is activated in an inflammatory environment by various pro-inflammatory cytokines, including interleukin (IL)-1β and tumor necrosis factor (TNF)-α (13, 14). Since these cytokines are mainly produced by multiple immune cells, including macrophages and CD4^+^ T lymphocytes, this establishes a tight reciprocal interaction between hPDL-MSCs and immune cells (15).

**Figure 1:**
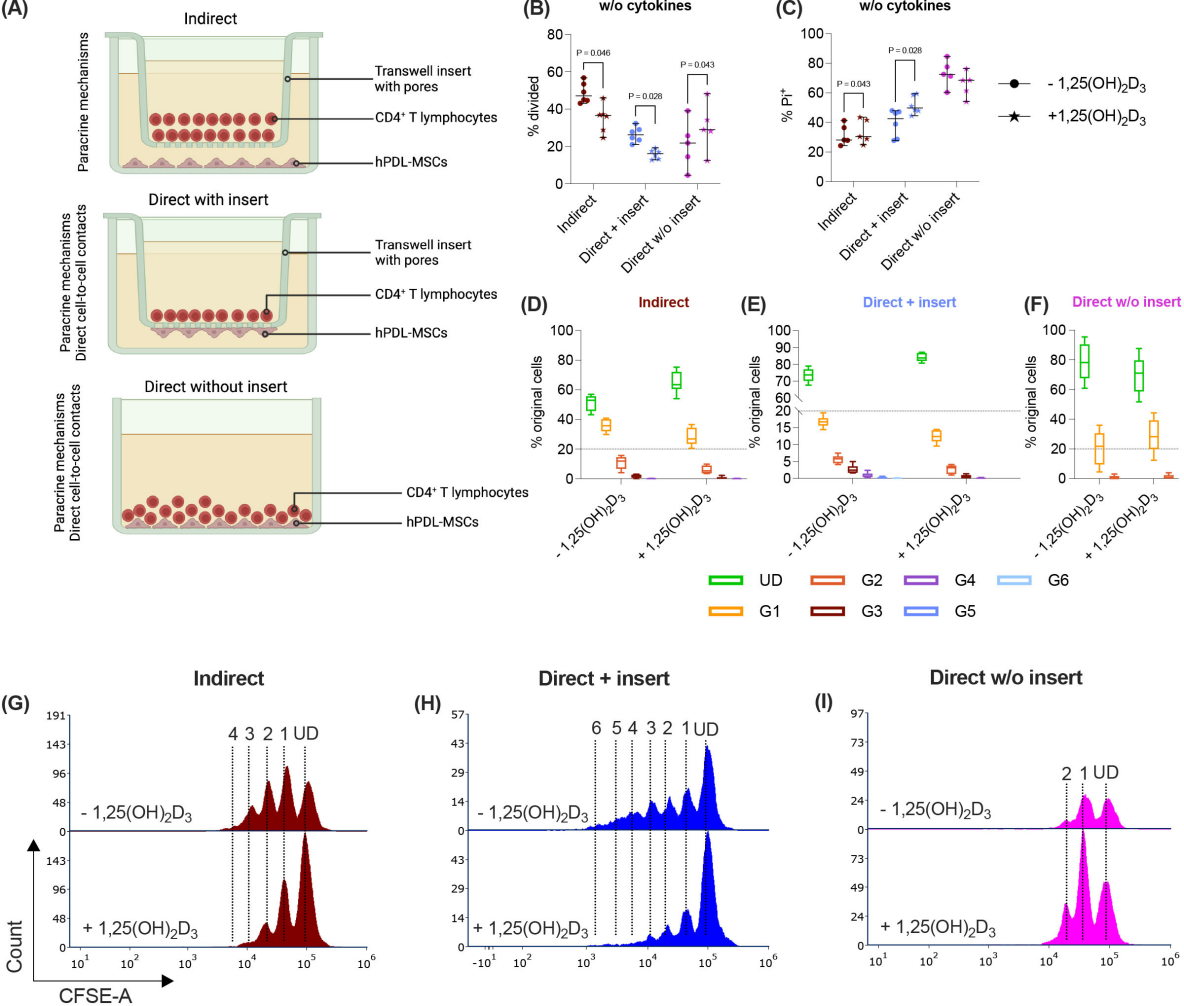
In the direct co-culture model without inserts, 1,25(OH)_2_D_3_ differently impacts CD4^+^ T lymphocyte proliferation and viability via hPDL-MSCs compared to the other models. Depending on the co-culture model **(A)**, hPDL-MSCs were seeded in 6-well plates or at the membrane’s underside of transwell inserts (TC) and were treated with 1,25(OH)_2_D_3_. CD4^+^ T lymphocytes were added either into the TC insert or directly to the hPDL-MSCs and were activated by phytohemagglutinin. After five days of incubation, CFSE and Pi stainings were analyzed by flow cytometry to determine the proliferation and viability of CD4^+^ T lymphocytes, respectively **(B-I)**. The % of original CD4^+^ T lymphocytes that have proliferated is presented in **(B)**, whereas the % of (Pi^+^) dead CD4^+^ T lymphocytes is shown in **(C)**. For the undivided (UD) and each divided (G1-G6) generation, the number of original CD4^+^ T lymphocytes is demonstrated as the percentage of the total original cell number **(D-F)**. **(G, H)** show representative histograms with absolute numbers of CD4^+^ T lymphocytes for each generation (UD and G1-G6). The data were obtained from five independent repetitions using hPDL-MSCs isolated from five distinct individuals. In **(B, C)**, single data points, the median, and the confidence intervals (95%) are presented. The data in **(D-F)** are presented as box-whisker plots, including the minimum and maximum values. Statistically significant differences are shown by providing P-values obtained using the Wilcoxon Test for pairwise comparison. **Figure 1A** was created with Biorender.

Most of the information about these bidirectional interactions is derived from two different *in vitro* models that co-culture hPDL-MSCs with immune cells (**Figure 1A**) (14, 16–19). The indirect co-culture model, which uses transwell inserts, segregates hPDL-MSCs and immune cells by a liquid-permeable membrane without any direct cell-co-tell contacts (16, 18, 20). This allows us to investigate the paracrine mechanisms exclusively between hPDL-MSCs and immune cells but hardly imitates the *in vivo* situation (12). In contrast, the direct co-culture model contains an intermixture of immune cells with the plastic-adherent hPDL-MSCs, considering paracrine and direct cell-to-cell contact mechanisms (17–19). Another version of the direct co-culture model uses transwell inserts with hPDL-MSCs seeded on the bottom side of the porous membrane and immune cells within the inserts (21). This allows easy separation of the different cell types for analyses but a limited direct interaction between hPDL-MSCs and immune cells via pores (12). All three co-culture models are suitable for discriminating between paracrine and direct cell-to-cell contact mechanisms. As shown by our recent study, the effects of hPDL-MSCs on the CD4^+^ T lymphocytes depend qualitatively and quantitatively on the co-culture model and inflammatory environment (22).

1,25-dihydroxyvitamin-D_3_ (1,25(OH)_2_D_3_), the biologically active vitamin D_3_ form (23, 24), is well known to have anti-inflammatory effects, directly influencing innate and adaptive immune cells (25). These anti-inflammatory properties are also indirectly executed via MSCs (20), which was demonstrated by Ghalavand et al. (26), who observed the anti-inflammatory effects of 1,25(OH)_2_D_3_-treated MSCs in an allergic asthma mouse model. It is well-established that 1,25(OH)_2_D_3_ inhibits the secretion of pro-inflammatory cytokines (e.g. IL-6, IL-8, and monocyte chemotactic protein-1 (MCP-1) in hPDL-MSCs in the presence of various stimuli, including *Porphyromonas gingivalis* (*P. gingivalis*)-derived lipopolysaccharide (LPS), heat-inactivated *P. gingivalis*, and IL-1β (27–30). Our previous study, using the indirect co-culture model and considering only paracrine mechanisms, demonstrated a significant inhibitory potential of 1,25(OH)_2_D_3_ on CD4^+^ T lymphocyte proliferation via TNF-α, or IL-1β-treated hPDL-MSCs (20). To the best of our knowledge, no other studies have discriminated the influence of 1,25(OH)_2_D_3_ on the paracrine and direct cell-to-cell contact mechanisms between hPDL-MSCs and immune cells using different co-culture models.

Hence, this study aimed to elucidate the impact of 1,25(OH)_2_D_3_ on the interaction between hPDL-MSCs and CD4^+^ T lymphocytes using the different *in vitro* co-culture models. We co-cultured activated CD4^+^ T lymphocytes with TNF-α or IL-1β-treated hPDL-MSCs in the presence or absence of 1,25(OH)_2_D_3_ and investigated CD4^+^ T lymphocyte proliferation, viability, and cytokine secretion as the main outcome parameters. Additionally, to determine the immunomodulatory mechanisms by which 1,25(OH)_2_D_3_ affects CD4^+^ T lymphocytes, the immunomediator gene expression of the differently co-cultured hPDL-MSCs was analyzed.

## 2 Material and methods

### 2.1 Ethical approval

The Ethics Committee of the Medical University of Vienna approved the isolation of primary hPDL-MSCs from extracted teeth and CD4^+^ T lymphocytes from whole blood samples (EK Nr.: 1694/2015, valid up to 10/2024). All procedures complied with the Declaration of Helsinki and the Medical University of Vienna’s Good Scientific Practice Guidelines. The informed written consent was obtained from each participant.

### 2.2 Primary hPDL-MSCs isolation and cultivation

Extracted third molars from 6 different individuals (five females and one male, aged between 18 and 27 years) were used to isolate hPDL-MSCs. The teeth were extracted from periodontally healthy individuals because of orthodontic reasons. The patients were informed and gave their written agreement before the extraction procedure. The PDL tissue was removed from the roots’ mid-third of the extracted teeth, minced into small pieces, and cultured until hPDL-MSCs had grown out of the tissue slices. PDL tissue slices and hPDL-MSCs were cultivated in Dulbecco’s Modified Eagle’s medium (DMEM, Sigma-Aldrich, St. Louis, MO, USA) supplemented with sodium bicarbonate, L-glutamine, 4500 mg/ml glucose, 50 µg/ml streptomycin (Gibco, Carlsbad, CA, USA), 100 U/ml penicillin (Gibco, Carlsbad, CA, USA), and 10% fetal bovine serum (FBS, Gibco, Carlsbad, CA, USA) at 37° Celsius, 5% CO_2_, and 95% humidity. When the hPDL-MSCs reached 80% confluency, the cells were passaged. Passages between five and seven were used for experiments.

Following the minimal criteria for MSCs from the ISCT (2), the expression of specific MSCs’ (CD29, CD90, CD105, and CD146) and the lack of hematopoietic (CD14, CD31, CD34, and CD45) surface markers were verified for each donor by immunostaining followed by flow cytometry and the osteogenic differentiation potential was investigated by Alizarin Red staining. The results of these verifications have already been published in our previous studies (14, 31, 32).

### 2.3 Allogeneic CD4^+^ T lymphocyte isolation and cultivation

Peripheral blood mononuclear cells (PBMCs) were extracted from venous whole blood, which was taken from one volunteer (systemically healthy, male, 31 years old) using the lithium- and heparin-containing VACUETTE^®^ blood collection system (Greiner Bio-one, Kremsmünster, Austria). After mixing the blood 1:1 with Hank’s Balanced Salt Solution (HBSS, Life Technologies, Carlsbad, CA, USA), the diluted blood was layered on top of Ficoll-Paque (GE Healthcare, Chicago, IL, USA) before performing a density gradient centrifugation. The isolated PBMCs were washed with HBSS once and were diluted in 1X phosphate-buffered saline (1X PBS). Primary CD4^+^ T lymphocytes were enriched by negative immunomagnetic selection using the MagniSort™ Human CD4^+^ T cell enrichment Kit (Invitrogen, Carlsbad, CA, USA) following the suppliers’ instructions. The purity of the enriched CD4^+^ T lymphocyte population was verified in our previous study (>90%) by CD4 immunostaining followed by flow cytometry (20).

The freshly isolated CD4^+^ T lymphocytes were immediately stained with carboxyfluorescein succinimidyl ester (CFSE) using the CellTrace™ CFSE Cell Proliferation Kit (Thermo Fisher Scientific, Waltham, MA, USA) following the manufacturer’s instructions. Briefly, CD4^+^ T lymphocytes were resuspended in 1xPBS supplemented with 5% FBS, reaching a final concentration of 1x10^6^ CD4^+^ T lymphocytes per ml. The CD4^+^ T lymphocytes were stained for five minutes at room temperature with 0.5 µl CFSE per ml to obtain a final working concentration of 2.5 µM. Subsequently, CD4^+^ T lymphocytes were washed by adding RPMI-1640 (Sigma-Aldrich, St. Louis, MO, USA), supplemented with L-glutamine, sodium bicarbonate, 50 µg/ml streptomycin, 100 U/ml penicillin, and 10% FBS. After centrifugation, the CFSE-stained CD4^+^ T lymphocytes were resuspended with 1 ml fully supplemented RPMI-1640 medium per 1x10^6^ CD4^+^ T lymphocytes, incubated for 10 minutes at 37° Celsius, and co-cultured with hPDL-MSCs in the different models at 37° Celsius, 5% CO_2_, and 95% humidity.

### 2.4 hPDL-MSCs and CD4^+^ T lymphocyte co-cultivation

hPDL-MSCs (2x10^4^/cm^2^) and allogeneic CD4^+^ T lymphocytes were co-cultured 1:4 using the following three co-culture models (**Figure 1A**):

Indirect co-culture model: 2.5x10^5^ hPDL-MSCs (2x10^4^ hPDL-MSCs/cm^2^) were seeded per well in 3 ml fully supplemented DMEM medium using 6-well plates for 24 hours. hPDL-MSCs were pre-treated with 100 nM 1,25(OH)_2_D_3_ in the absence or presence of 5 ng/ml IL-1β, or 10 ng/ml TNF-α (both from Peprotech, London, United Kingdom) using DMEM medium without FBS. hPDL-MSCs without 1,25(OH)_2_D_3_ served as control. After an additional 48 hours of incubation, 1x10^6^ freshly isolated and CFSE-stained CD4^+^ T lymphocytes were added in transwell (TC) inserts (pore size: 0.4 µm; Sarstedt, Biedermannsdorf, Austria) using 1 ml fully supplemented RPMI-1640 medium per insert.

Direct co-culture with insert: 1x10^5^ hPDL-MSCs (2x10^4^ hPDL-MSCs/cm^2^) were seeded at the membrane’s underside of TC inserts. After 24 hours of incubation, the hPDL-MSCs-containing TC inserts were transferred to 6-well plates, and hPDL-MSCs were pre-treated as described above. After 48 hours of incubation, 4x10^5^ freshly isolated and CFSE-stained CD4^+^ T lymphocytes were added to the TC inserts using 1 ml of a fully supplemented RPMI-1640 medium per insert.

Direct co-culture without insert: 2.5x10^5^ hPDL-MSCs (2x10^4^ hPDL-MSCs/cm^2^) were seeded in 6-well plates for 24 hours, as described for the indirect co-culture model. hPDL-MSCs were pre-stimulated, as mentioned above. After 48 hours, 1x10^6^ freshly isolated and CFSE-stained CD4^+^ T lymphocytes were added per well directly to the hPDL-MSCs without any insert, using 2ml complete RPMI-1640 medium per well.

Regardless of the co-culture model, CD4^+^ T lymphocyte proliferation was induced with 10 µg/ml phytohemagglutinin-L (PHA-L, Thermo Fisher Scientific, Waltham, MA, USA). PHA-L activated and non-activated CD4^+^ T lymphocytes without hPDL-MSCs served as control. hPDL-MSCs were re-stimulated as described above using 2 ml fully supplemented RPMI-1640 medium per well. After five days of incubation, CD4^+^ T lymphocyte proliferation and viability were analyzed using the CFSE, and propidium iodide (Pi, both from Thermo Fisher Scientific, Waltham, MA, USA) staining, followed by flow cytometry analysis. CD4^+^ T lymphocyte-conditioned media were harvested, and cytokine levels were determined by multiplexing (LEGENDplex™, BioLegend, San Diego, CA, USA). Immunomediator gene expression in co-cultured hPDL-MSCs was determined by quantitative polymerase chain reaction (qPCR).

### 2.5 Analysis

#### 2.5.1 CD4^+^ T lymphocytes – CFSE proliferation and Pi viability assay

After five days of incubation, CFSE-stained CD4^+^ T lymphocytes were harvested and washed with 3% bovine serum albumin (BSA, Merck Millipore, Burlington, USA; dissolved in 1X PBS + 0.09% sodium azide). Dead CD4^+^ T lymphocytes were stained by adding 20 µg/ml propidium iodide (Pi, Thermo Fisher Scientific, Waltham, MA, USA), immediately followed by flow cytometer acquisition using the Attune Nxt Acoustic Focusing flow cytometer (Thermo Fisher Scientific, Waltham, MA, USA). Both dyes were excited at 488nm using the blue laser. The light emitted by CFSE and Pi was detected by the BL1 and BL2 channels, respectively. Unlabelled CFSE- and Pi-single labeled controls, containing a 1:1 mixture of living and dead CD4^+^ T lymphocytes, were prepared for compensation. In total, 20,000 events were acquired per experimental sample. After excluding cell debris and co-incidence events, the percentage of Pi^+^ CD4^+^ T lymphocytes (% of dead cells) was determined. Only living (Pi^-^) CD4^+^ T lymphocytes were included in the proliferation analysis. The FCS Express 7 software (*De novo* Software by Dotmatics, Pasadena, CA, USA) was used for analysis and to calculate the % of Pi^+^ CD4^+^ T lymphocytes, the % of original CD4^+^ T lymphocytes that have proliferated (% divided), the number of original CD4^+^ T lymphocytes as the % of the total original cell number per generation, and the absolute numbers of CD4^+^ T lymphocytes for each generation.

#### 2.5.2 CD4^+^ T lymphocytes – cytokine multiplexing

The conditioned medium was harvested and stored at -80° Celsius, five days after starting the co-cultivation. The concentrations of 12 different Th cytokines (IL-2, IL-4, IL-5, IL-6, IL-9, IL-10, IL-13, IL-17A, IL-17F, IL-22, TNF-α, and IFN-γ) were simultaneously measured in the conditioned media by the LEGENDplex™ Multi-Analyte Flow Assay Kit (12-plex Human Th Cytokine Panel, BioLegend, San Diego, CA, USA) using the V-bottom plates. The samples were acquired by the Attune Nxt Acoustic Focusing flow cytometer (Thermo Fisher Scientific, Waltham, MA, USA). The photomultiplier tube (PMT) voltages were set in accordance with the manufacturer’s recommendations, and 3,600 beads were acquired in total. The LEGENDplex™ data analysis software (BioLegend, San Diego, CA, USA) was used for data analysis according to the suppliers’ instructions. The cytokine concentrations were calculated by five-parameter logistic standard curves and were normalized to the total CD4^+^ T lymphocyte cell number per sample, which was ascertained by Neubauer-improved cell counting chambers (NanoEnTek, Soul, South Korea) after five days of co-cultivation.

#### 2.5.3 hPDL-MSCs – immunomediator gene expression analysis

Five days after starting the co-cultivation, hPDL-MSCs were lysed, followed by complementary DNA (cDNA) synthesis, and qPCR using the TaqMan Gene Expression Cells-to-CT kit (Applied Biosystems, Foster City, CA, USA) in compliance with the manufacturer’s manual. In brief, after lysing hPDL-MSCs, mRNA was reverse transcribed into cDNA by heating the samples up to 37° Celsius for one hour, followed by 95° Celsius for five minutes using the Primus 96 advanced thermocycler (PeqLab/VWR, Darmstadt, Germany). qPCR was performed on a QuantStudio 3 device (Applied Biosystems, Foster City, CA, USA), heating the samples to 95° Celsius for ten minutes. This was followed by 50 cycles of heating the samples to 95° Celsius for 15 seconds and cooling them to 60° Celsius for one minute. The amplification and detection of immunomodulator-specific cDNA was conducted in paired reactions by using the following TaqMan Gene Expression Assays (all from Applied Biosystems, Foster City, CA, USA): Hs00984148_m1 (*IDO-1*), Hs00153133_m1 (*PTGS-2*), Hs00200180_m1 (*TSG-6*), Hs00228839_m1 (*CD273*), Hs00204257_m1 (*CD274*), and Hs99999905_m1 (*GAPDH)*. The housekeeping gene glyceraldehyde-3-phosphate dehydrogenase (*GAPDH*) was included as an internal reference. Determined cycle threshold (Ct) values were normalized to *GAPDH* (ΔCt) and the appropriate controls (ΔΔCt) within each co-culture model. The 2^-ΔΔCt^ formula was used to calculate the n-fold expression of the target genes compared to the relevant controls (n-fold expression = 1).

#### 2.5.4 Statistical analysis

All statistical analysis was conducted using the SPSS Statistics software (version 26.0, IBM, Armonk, USA). The Kolmogorov-Smirnov Test checked the normal distribution of data. Due to non-parametric and paired data, the Friedman Test was used for multiple comparisons, followed by the Wilcoxon Test for pairwise comparisons. P-values < 0.05 were statistically significant. All data were gained from at least five experimental repetitions, using hPDL-MSCs from a different individual for each repetition.

## 3 Results

### 3.1 Depending on the co-culture model, 1,25(OH)_2_D_3_ differently affects CD4^+^ T lymphocyte proliferation and viability via hPDL-MSCs

Paracrine and direct cell-to-cell contact mechanisms contribute to the immunomodulatory activities of hPDL-MSCs against CD4^+^ T lymphocytes (4, 12). Therefore, we used three different co-culture setups to investigate how 1,25(OH)_2_D_3_ influences the CD4^+^ T lymphocyte proliferation (**Figures 1B, D-I**) and viability (**Figure 1C**) via hPDL-MSCs. The data show that 1,25(OH)_2_D_3_ significantly reduced the CD4^+^ T lymphocyte proliferation (**Figure 1B**) via hPDL-MSCs in the indirect and direct co-culture with inserts. Lower proliferation is also indicated by a higher % of original CD4^+^ T lymphocytes of the divided generations (**Figures 1D, E**) and a lower number of divided generations (**Figures 1G, H**) in the presence of 1,25(OH)_2_D_3_. In the direct co-culture without inserts, 1,25(OH)_2_D_3_ significantly increased the % of divided CD4^+^ T lymphocytes and the % of original CD4^+^ T lymphocytes of the divided generations (**Figures 1F, I**) but showed no changes in the number of divided CD4^+^ T lymphocyte generations.

Additionally, 1,25(OH)_2_D_3_ significantly increased the percentage of Pi^+^ CD4^+^ T lymphocytes in the indirect and direct co-culture model with inserts. In contrast, the direct co-culture without inserts demonstrated a decrease in Pi^+^ CD4^+^ T lymphocytes in the presence of 1,25(OH)_2_D_3_, however, without significance (**Figure 1C**). These data indicate that the hPDL-MSCs’ based effects of 1,25(OH)_2_D_3_ on CD4^+^ T lymphocyte proliferation and viability depend on the used co-culture model.

### 3.2 In different co-culture setups, 1,25(OH)_2_D_3_ predominantly suppresses the cytokine secretion in CD4^+^ T lymphocytes to varying degrees through hPDL-MSCs

**Figure 2:**
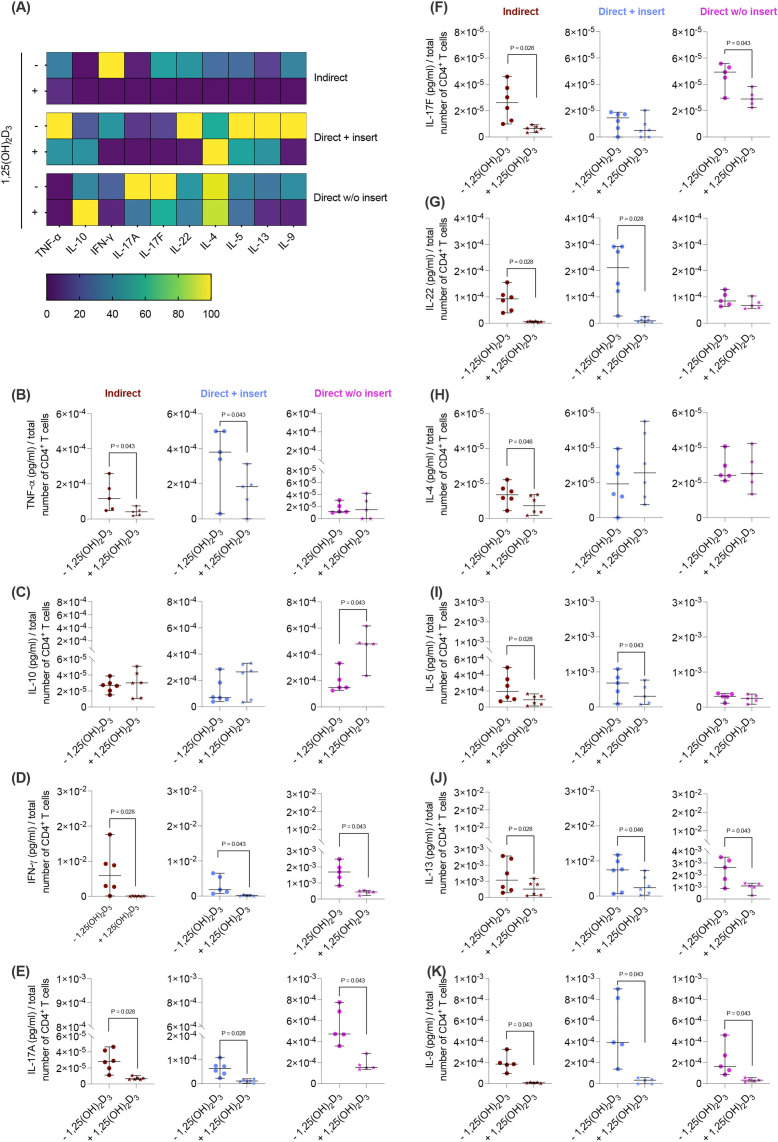
1,25(OH)_2_D_3_ affects the cytokine production of CD4^+^ T lymphocytes through hPDL-MSCs regardless of the co-culture models. After five days of co-cultivation, the conditioned medium of CD4^+^ T lymphocytes was harvested, and the levels of secreted cytokines were determined by a bead-based multiplexing assay. The heatmap represents the normalized data as a percentage **(A)**. In **(B-K)**, the measured cytokine concentrations (pg/ml) were normalized to the appropriate total CD4^+^ T lymphocyte number per group. In **(B-K)**, single data points, the median, and the confidence intervals (95%) are presented. The data were obtained from five to six independent repetitions using hPDL-MSCs isolated from five to six distinct individuals. Statistically significant differences are shown by providing P-values obtained using the Wilcoxon Test for pairwise comparison.

Next, we elucidated how the different co-culture models impact the effects of 1,25(OH)_2_D_3_ on the cytokine secretion by CD4^+^ T lymphocytes in the presence of hPDL-MSCs. 1,25(OH)_2_D_3_ predominantly diminished the levels of all cytokines (**Figures 2A, B, D-K**) except IL-10 (**Figure 2C**). However, the degree of these suppressions varies between the different co-culture setups. IFN-γ (**Figure 2D**), IL-17A (**Figure 2E**), IL-13 (**Figure 2J**), and IL-9 (**Figure 2K**) levels were significantly reduced by 1,25(OH)_2_D_3_ in all three co-culture model types (IFN-γ; 108-fold, 21-fold, 3.9-fold; IL-17A: 4.1-fold, 5.3-fold, 3.0-fold; IL-13: 2.4-fold, 2.1-fold, 2.4-fold; IL-9: 22-fold, 19-fold, 6.3-fold). A significant reduction of TNF-α (3.1-fold, 2.2-fold; **Figure 2B**), IL-22 (15-fold, 17-fold; **Figure 2G**), and IL-5 (2.6-fold, 1.7-fold; **Figure 2I**) was observed in the indirect and direct co-culture models with inserts, whereas IL-17F (**Figure 2F**) was significantly decreased in the indirect (4.2-fold) and direct co-culture without inserts (1.6-fold). IL-4 (**Figure 2H**) was significantly reduced only in the indirect co-culture model type (1.8-fold). In contrast, IL-10 (**Figure 2C**) levels were significantly increased by 1,25(OH)_2_D_3_ after direct co-cultivation without inserts (2.4-fold). Based on the data presented, it appears that the degree of the hPDL-MSCs-based cytokine inhibition in CD4^+^ T lymphocytes by 1,25(OH)_2_D_3_ depends on the co-culture setups.

### 3.3 The effects of 1,25(OH)_2_D_3_ via IL-1β-treated hPDL-MSCs on CD4^+^ T lymphocyte proliferation and viability varies, depending on the co-culture model

**Figure 3:**
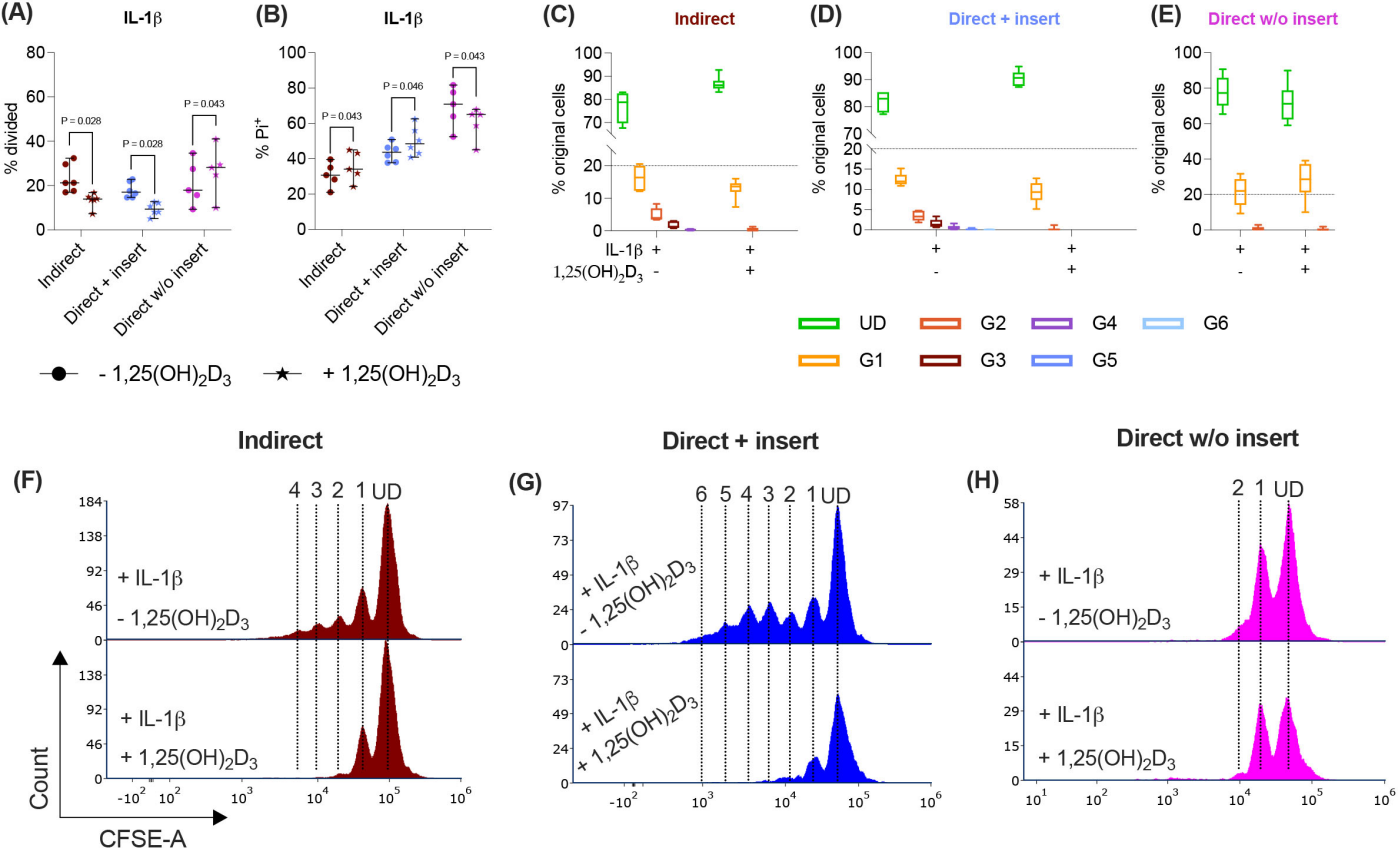
In the direct co-culture model without inserts, 1,25(OH)_2_D_3_ affects CD4^+^ T lymphocyte proliferation and viability via IL-1β-treated hPDL-MSCs differently compared to the other models. After five days of co-cultivation, CFSE and Pi stainings were analyzed by flow cytometry to determine the proliferation and viability of CD4^+^ T lymphocytes, respectively **(A-H)**. The % of original CD4^+^ T lymphocytes that have proliferated is presented in **(A)**, whereas the % of (Pi^+^) dead CD4^+^ T lymphocytes is shown in **(B)**. For the undivided (UD) and each divided (G1-G6) generation, the number of original CD4^+^ T lymphocytes is demonstrated as the percentage of the total original cell number **(C-E)**. **(F-H)** show representative histograms with absolute numbers of CD4^+^ T lymphocytes for each generation (UD and G1-G6). The data were obtained from five to six independent repetitions using hPDL-MSCs isolated from five to six distinct individuals. In **(A, B)**, single data points, the median, and the confidence intervals (95%) are presented. The data in **(C-E)** are presented as box-whisker plots, including the minimum and maximum values. Statistically significant differences are shown by providing P-values obtained using the Wilcoxon Test for pairwise comparison.

Various cytokines, including IL-1β, significantly boost the immunomodulatory activities of hPDL-MSCs (13, 14). Therefore, we added IL-1β to the differently co-cultured hPDL-MSCs in the presence and absence of 1,25(OH)_2_D_3_ and determined CD4^+^ T lymphocyte proliferation (**Figures 3A, C-H**) and viability (**Figure 3B**). In the indirect and direct co-culture models with inserts, 1,25(OH)_2_D_3_ significantly reduced CD4^+^ T lymphocyte proliferation in the presence of IL-1β-treated hPDL-MSCs (**Figure 3A**). This was endorsed by a reduced % of original CD4^+^ T lymphocytes in the divided generations (**Figures 3C, D**), and a diminished number of divided generations in the presence of 1,25(OH)_2_D_3_ (**Figures 3F, G**). In contrast, 1,25(OH)_2_D_3_ significantly increased CD4^+^ T lymphocyte proliferation when directly co-cultured with IL-1β-treated hPDL-MSCs without inserts (**Figure 3B**). This was also shown by a decreased and increased % of original CD4^+^ T lymphocytes in the undivided and divided cell generations, respectively (**Figure 3E**). No changes were observed in the number of divided CD4^+^ T lymphocyte generations in the direct co-culture model without inserts (**Figure 3H**).

Indirect and direct co-culturing CD4^+^ T lymphocytes in inserts with IL-1β-treated hPDL-MSCs caused a significant increase in the percentage of dead CD4^+^ T lymphocytes by 1,25(OH)_2_D_3_. In the direct co-culture without inserts, 1,25(OH)_2_D_3_ significantly reduced the % of Pi^+^ CD4^+^ T lymphocytes via IL-1β-treated hPDL-MSCs (**Figure 3B**). Together, these data demonstrate a dependency of 1,25(OH)_2_D_3_ effects on the used co-culture model in the presence of exogenous IL-1β.

### 3.4 The effect of 1,25(OH)_2_D_3_ on the cytokine secretion in CD4^+^ T lymphocytes through IL-1β-treated hPDL-MSCs partially depends on the co-culture setup

**Figure 4:**
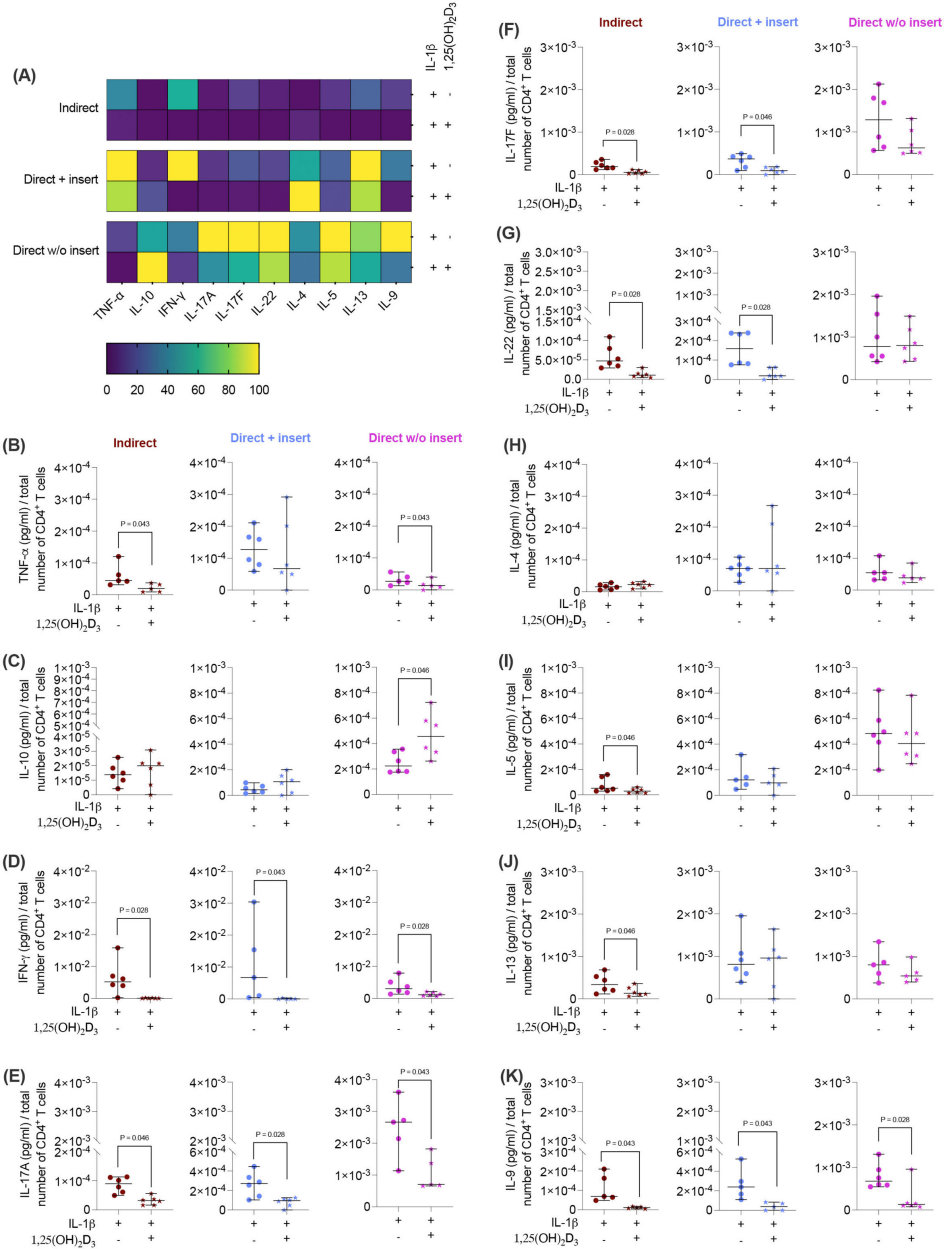
1,25(OH)_2_D_3_ influences the production of some cytokines by CD4^+^ T lymphocytes through IL-1β-treated hPDL-MSCs, and this effect partially depends on the co-culture models. After five days of co-cultivation, the conditioned medium of CD4^+^ T lymphocytes was harvested, and the levels of secreted cytokines were determined by a bead-based multiplexing assay. The heatmap represents the normalized data as a percentage **(A)**. In **(B-K)**, the measured cytokine concentrations (pg/ml) were normalized to the appropriate total CD4^+^ T lymphocyte number per group. In **(B-K)**, single data points, the median, and the confidence intervals (95%) are presented. The data were obtained from five to six independent repetitions using hPDL-MSCs isolated from five to six distinct individuals. Statistically significant differences are shown by providing P-values obtained using the Wilcoxon Test for pairwise comparison.

Next, we investigated how the cytokine secretion in CD4^+^ T lymphocytes (**Figures 4A-K**) is influenced by 1,25(OH)_2_D_3_ via IL-1β-treated hPDL-MSCs using different co-culture models. 1,25(OH)_2_D_3_ largely reduced the concentrations of most cytokines (**Figures 4A, B, D-G, I-K**) except IL-10 and IL-4 (**Figures 4C, H**). Interestingly, the degree of these inhibitions varies between the different co-culture setups. IFN-γ (**Figure 4D**), IL-17A (**Figure 4E**), and IL-9 (**Figure 4K**) levels were significantly diminished by 1,25(OH)_2_D_3_ after co-cultivation with IL-1β-treated hPDL-MSCs in all three model types (IFN-γ: 114-fold, 163-fold, 2.8-fold; IL-17A: 2.7-fold, 3.3-fold, 2.3-fold; IL-9: 10-fold, 7.0-fold, 3.0-fold). A significant reduction of IL-17F (3.4-fold, 3.2-fold; **Figure 4F**), and IL-22 (4.4-fold, 5.2-fold; **Figure 4G**) were detected in the indirect and direct models with inserts, whereas TNF-α (**Figure 4B**) concentrations were significantly reduced by 1,25(OH)_2_D_3_ in the indirect (2.8-fold) and direct model without inserts (2.0-fold). IL-5 (2.4-fold; **Figure 4I**) and IL-13 (2.1-fold; **Figure 4J**) significantly decreased only in the indirect co-culture model type. In contrast, IL-10 (**Figure 4C**) levels were significantly enhanced by 1,25(OH)_2_D_3_ after direct co-cultivation with IL-1β-treated hPDL-MSCs without inserts (1.8-fold). No changes were observed for IL-4 (**Figure 4H**). These data indicate that the hPDL-MSCs-based cytokine suppression in CD4^+^ T lymphocytes by 1,25(OH)_2_D_3_ depends on the co-culture setup in the presence of exogenous IL-1β.

### 3.5 The impact of 1,25(OH)_2_D_3_ via TNF-α-stimulated hPDL-MSCs on CD4^+^ T lymphocyte proliferation and viability differs, depending on the co-culture model

**Figure 5:**
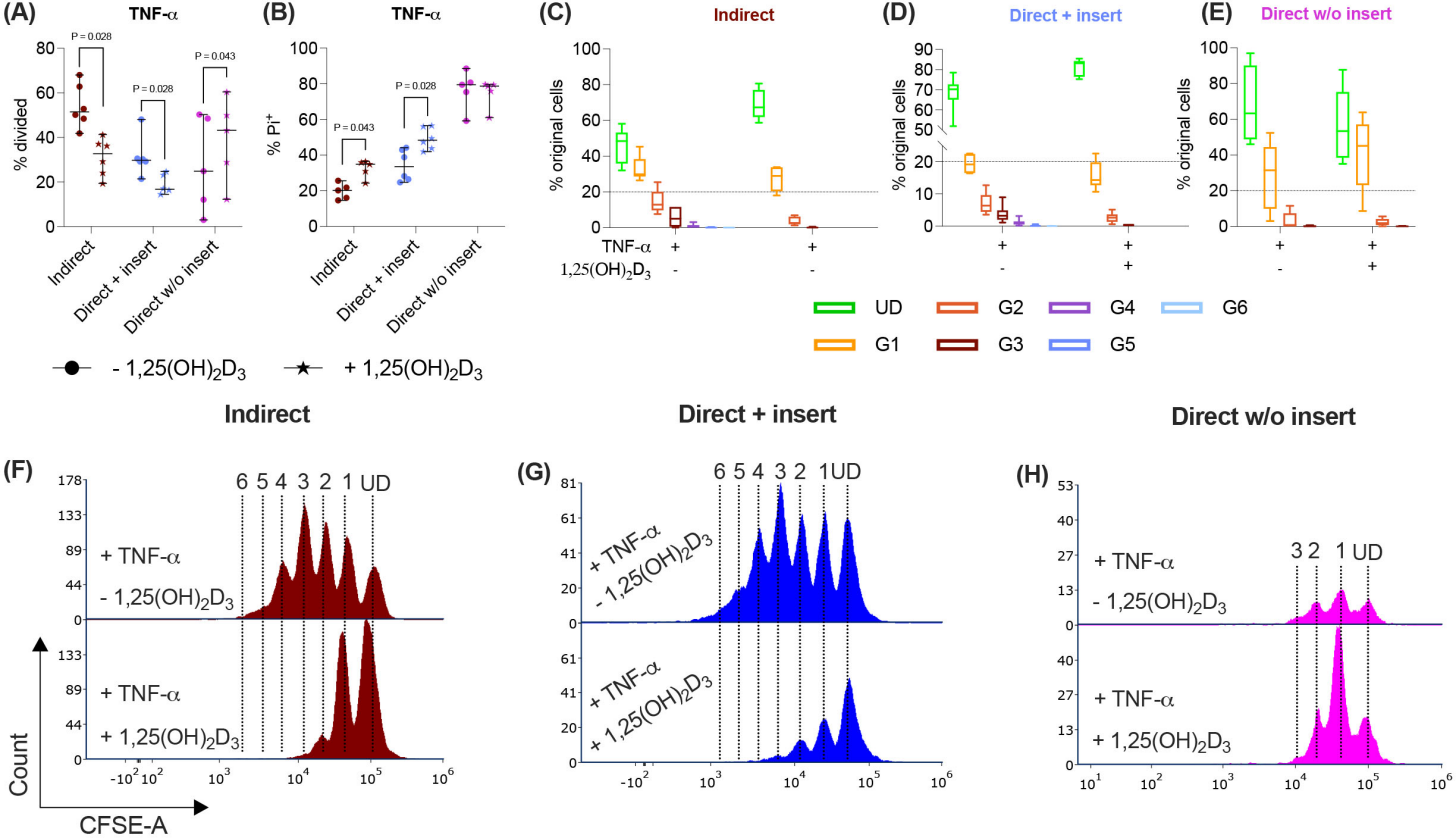
In the direct co-culture model without insert, 1,25(OH)_2_D_3_ influences CD4^+^ T lymphocyte proliferation and viability via TNF-treated hPDL-MSCs differently compared to the other models. After five days of co-cultivation, CFSE and Pi stainings were analyzed by flow cytometry to determine the proliferation and viability of CD4^+^ T lymphocytes, respectively **(A-H)**. The % of original CD4^+^ T lymphocytes that have proliferated is shown in **(A)**, whereas the % of (Pi^+^) dead CD4^+^ T lymphocytes is presented in **(B)**. For the undivided (UD) and each divided (G1-G6) generation, the number of original CD4^+^ T lymphocytes is demonstrated as the percentage of the total original cell number **(C-E)**. **(F-H)** show representative histograms with absolute numbers of CD4^+^ T lymphocytes for each generation (UD and G1-G6). The data were obtained from five to six independent repetitions using hPDL-MSCs isolated from five to six distinct individuals. In **(A, B)**, single data points, the median, and the confidence intervals (95%) are presented. The data in **(C-E)** are presented as box-whisker plots, including the minimum and maximum values. Statistically significant differences are shown by providing P-values obtained using the Wilcoxon Test for pairwise comparison.

Besides IL-1β, TNF-α also increases the immunomodulatory potential of hPDL-MSCs against CD4^+^ T lymphocytes (13, 14). Therefore, we have added TNF-α to the differently co-cultured hPDL-MSCs in the presence or absence of 1,25(OH)_2_D_3_ and determined the CD4^+^ T lymphocyte proliferation (**Figures 5A, C-H**) and viability (**Figure 5B**). 1,25(OH)_2_D_3_ significantly reduced the CD4^+^ T lymphocyte proliferation when indirectly and directly co-cultured in inserts with TNF-α-treated hPDL-MSCs (**Figure 5A**). This was confirmed by a reduced % of original CD4^+^ T lymphocytes in all divided generations (**Figures 5C-D**) and fewer divided CD4^+^ T lymphocyte generations (**Figures 5F-G**) in the presence of 1,25(OH)_2_D_3_. In the direct co-culture without insert, 1,25(OH)_2_D_3_ significantly increased the CD4^+^ T lymphocyte proliferation in the presence of 1,25(OH)_2_D_3_ (**Figure 5A**), showing a decreased and increased % of original CD4^+^ T lymphocytes in the undivided and divided cell generations, respectively (**Figure 5E**). However, no changes in the number of divided CD4^+^ T lymphocyte generations were observed (**Figure 5H**).

The indirect and direct co-culture of CD4^+^ T lymphocytes in inserts with TNF-α-triggered hPDL-MSCs caused a significant rise in the percentage of dead CD4^+^ T lymphocytes by 1,25(OH)_2_D_3_ (**Figure 5B**). In the direct co-culture without inserts, 1,25(OH)_2_D_3_ reduced the % of dead CD4^+^ T lymphocytes via TNF-α-treated hPDL-MSCs, however, without any significance (**Figure 5B**). Together, these data indicate that the observed 1,25(OH)_2_D_3_ effects via TNF-α-stimulated hPDL-MSCs depend on the used co-culture models.

### 3.6 The effect of 1,25(OH)_2_D_3_ on the cytokine secretion in CD4^+^ T lymphocytes through TNF-α-stimulated hPDL-MSCs minimally depends on the co-culture setup

**Figure 6:**
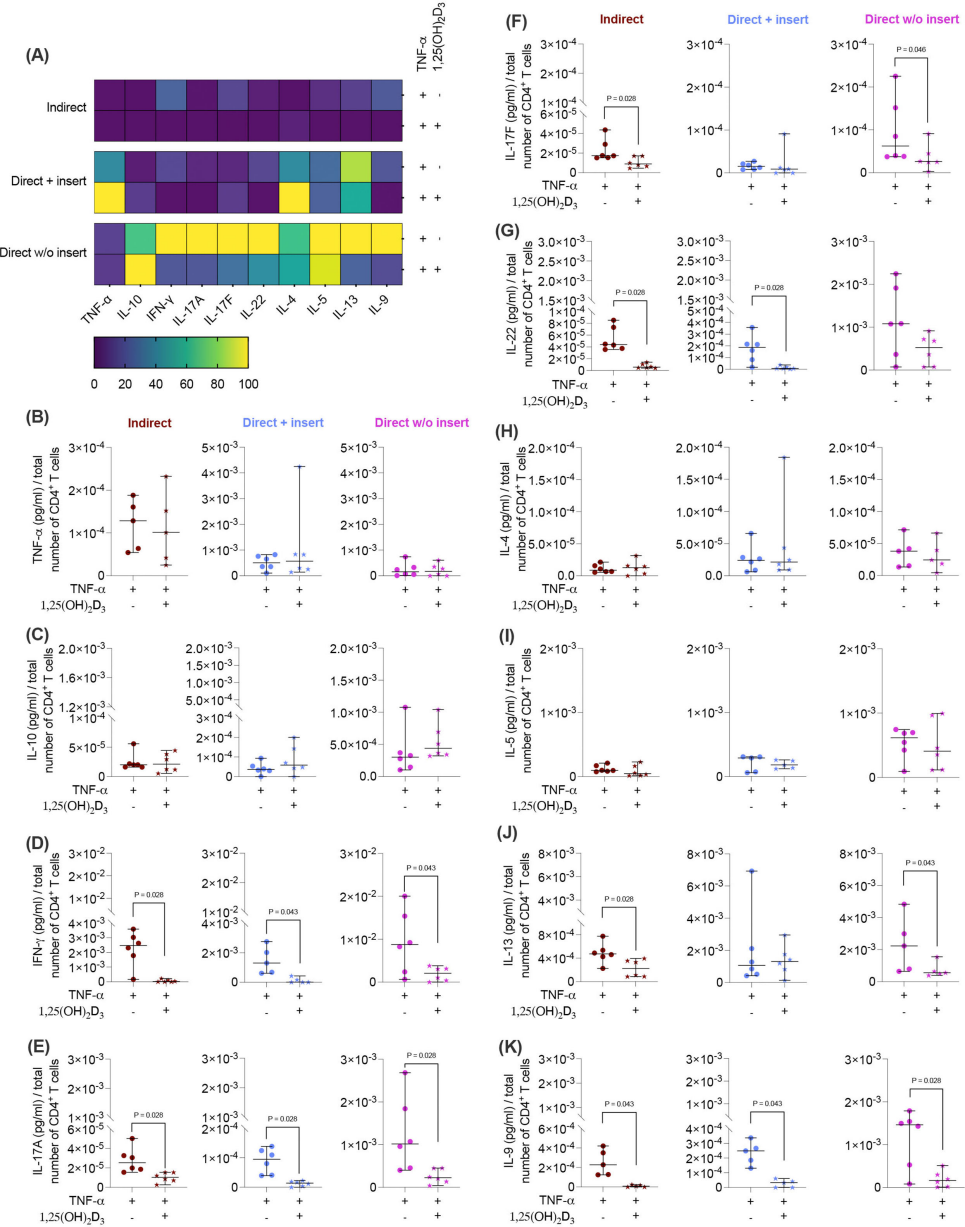
1,25(OH)_2_D_3_ partly affects the cytokine production of CD4^+^ T lymphocytes through TNF-α-treated hPDL-MSCs, depending on the co-culture models. After five days of co-cultivation, the conditioned medium of CD4^+^ T lymphocytes was harvested, and a bead-based multiplexing assay determined the levels of secreted cytokines. The heatmap represents the normalized data as a percentage **(A)**. In **(B-K)**, the measured cytokine concentrations (pg/ml) were normalized to the appropriate total CD4^+^ T lymphocyte number per group. In **(B-K)**, single data points, the median, and the confidence intervals (95%) are presented. The data were obtained from five to six independent repetitions using hPDL-MSCs isolated from five to six distinct individuals. Statistically significant differences are shown by providing P-values obtained using the Wilcoxon Test for pairwise comparison.

Next, we investigated how the cytokine secretion in CD4^+^ T lymphocytes (**Figures 6A-K**) is influenced by 1,25(OH)_2_D_3_ via TNF-α-treated hPDL-MSCs using different co-culture models. 1,25(OH)_2_D_3_ significantly decreased IFN-γ (**Figure 6D**), IL-17A (**Figure 6E**), and IL-9 (**Figure 6K**) in all three co-culture model types. The degree of these suppressions differed depending on the co-culture setup (IFN-γ: 37-fold, 12-fold, 4.9-fold; IL-17A: 2.7-fold, 7.2-fold, 4.8-fold; IL-9: 25-fold, 8.6-fold, 5.8-fold). IL-17F (2.2-fold, 2.7-fold; **Figure 6F**) and IL-13 (2.1-fold, 3.2-fold; **Figure 6J**) levels were significantly reduced in the indirect and direct co-culture model without inserts, whereas IL-22 (**Figure 6G**) was significantly diminished in the indirect (7.1-fold) and direct model with inserts (13-fold). In contrast, the production of several cytokines, particularly TNF-α (**Figure 6B**), IL-10 (**Figure 6C**), IL-4 (**Figure 6H**), and IL-5 (**Figure 6I**), was not affected by 1,25(OH)_2_D_3_ in any of the co-culture models. Together, these data indicate that in the presence of TNF-treated hPDL-MSCs, the partly cytokine inhibition in CD4^+^ T lymphocytes by 1,25(OH)_2_D_3_ exhibits a minimal dependency on the co-culture setup.

### 3.7 1,25(OH)_2_D_3_ mainly inhibits the gene expression of soluble immunomediators in hPDL-MSCs to a different extent depending on the co-culture model and the absence or presence of exogenous cytokines

**Figure 7:**
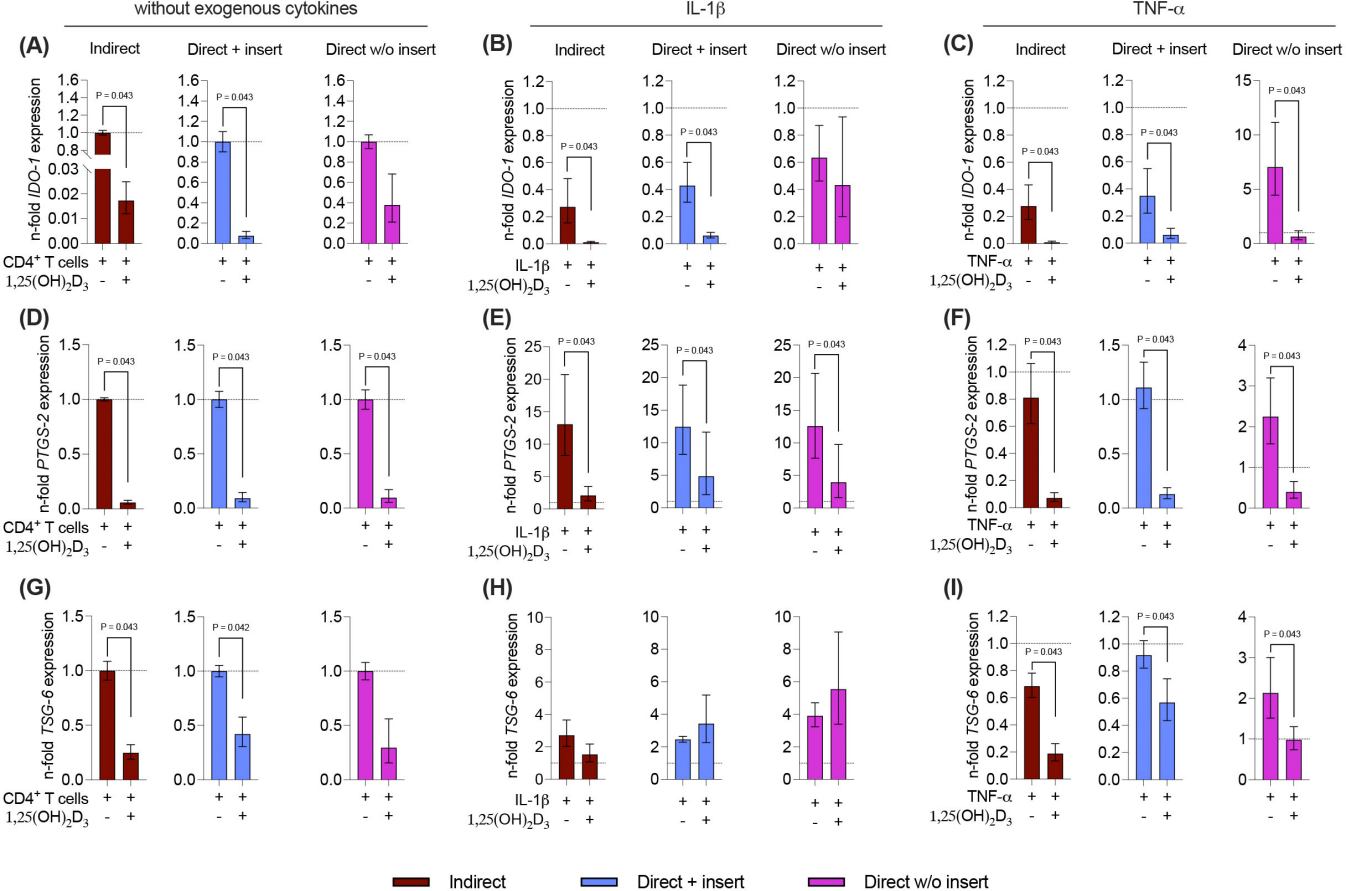
1,25(OH)_2_D_3_ mainly reduces the gene expression of the soluble immunomediators in hPDL-MSCs independently from the co-culture models. After five days of co-cultivation, basal, IL-1β-, or TNF-α-induced IDO-1 **(A-C)**, PTGS-2 **(D-F)**, and TSG-6 **(G-I)** gene expression levels were determined in co-cultured hPDL-MSCs using qPCR. The y-axes show the n-fold expression compared to the appropriate controls within the different co-culture models. hPDL-MSCs co-cultured with CD4^+^ T lymphocytes in the absence of any cytokine and 1,25(OH)_2_D_3_ served as controls (n-fold expression = 1; shown by dotted lines). GAPDH was used as a reference gene. The data are presented as mean +/- standard error of the mean (S.E.M.). The data were obtained from five independent experiments with two technical replicates per experiment, using hPDL-MSCs from five distinct individuals. Statistically significant differences are shown by providing P-values obtained using the Wilcoxon Test for pairwise comparison.

Due to the tight reciprocal interaction between hPDL-MSCs and CD4^+^ T lymphocytes, it is evident that CD4^+^ T lymphocytes should impact the immunomediator gene expression in the differently co-cultured hPDL-MSCs (15). Therefore, it is essential to investigate how 1,25(OH)_2_D_3_ influences the basal, IL-1β, or TNF-α-induced gene expression of soluble immunomediators in hPDL-MSCs in the different co-culture settings (**Figures 7A-I**). Regardless of the co-culture model, the basal, IL-1β, or TNF-α-induced gene expression of all soluble immunomediators (**Figures 7A-I**) was reduced by 1,25(OH)_2_D_3_, but quantitative differences between distinct models and inflammatory environment were observed. A significant reduction of *IDO-1* was observed in the indirect and direct co-culture model with inserts, in the absence of any exogenous cytokine (indirect: 58.8-fold decrease; direct with insert: 13.5-fold decrease; **Figure 7A**), in the presence of IL-1β (indirect: 24.7-fold decrease; direct with insert: 7.2-fold decrease; **Figure 7B**), or TNF-α (indirect: 30-fold decrease; direct with insert: 5.7-fold decrease; **Figure 7C**). Additionally, the TNF-α-induced *IDO-1* gene expression was significantly decreased in the direct co-culture without inserts (11-fold decrease; **Figure 7C**). *PTGS-2* gene expression was significantly reduced by 1,25(OH)_2_D_3_ independently from the used co-culture model and exogenous cytokines (**Figures 7D-F**). However, the extent of the decline differed between 17.2-fold, 10-9-fold, and 10.5-fold in the indirect, and direct co-culture with insert and the direct co-culture without insert, respectively, in the absence of exogenous cytokines (**Figure 7D**). In the presence of exogenous IL-1β, the extent of the decrease varied between 6.3-fold, 2.6-fold, and 3-2-fold in the indirect co-culture, the direct co-culture with insert, and the direct co-culture without insert, respectively (**Figure 7E**). The extent of the decrease of TNF-α-induced *PTGS-2* gene expression ranged between 11.4-fold, 8.7-fold, and 5.6-fold in the indirect co-culture, the direct co-culture with insert, and the direct co-culture without inserts, respectively (**Figure 7F**). In the indirect and direct co-culture model with inserts, 1,25(OH)_2_D_3_ significantly diminished the basal (indirect: 4-fold decrease; direct with insert: 2.3-fold decrease) and TNF-α-induced (indirect: 3.7-fold decrease; direct with insert: 1.6-fold decrease) *TSG-6* gene expression (**Figures 7G, I**). In the presence of TNF-α, 1,25(OH)_2_D_3_ also declined *TSG-6* gene expression in the direct co-culture model without insert (2.1-fold decrease; **Figure 7I**). The IL-1β-induced *TSG-6* gene expression was reduced by 1,25(OH)_2_D_3_ in the indirect co-culture model, whereas in the two direct co-culture models, an increase was observed (**Figure 7H**). However, no significances were detected in the presence of exogenous IL-1β. Together, these data indicate that the effects of 1,25(OH)_2_D_3_ on *IDO-1, PTGS-2*, and *TSG-6* gene expression levels in hPDL-MSCs are mainly suppressive, but the extent of these suppressions in some cases varies between the co-culture models and the present exogenous cytokines.

### 3.8 1,25(OH)_2_D_3_ partly inhibits the gene expression of membrane-bound immunomediators in hPDL-MSCs depending on the co-culture model and the presence of exogenous cytokines

**Figure 8:**
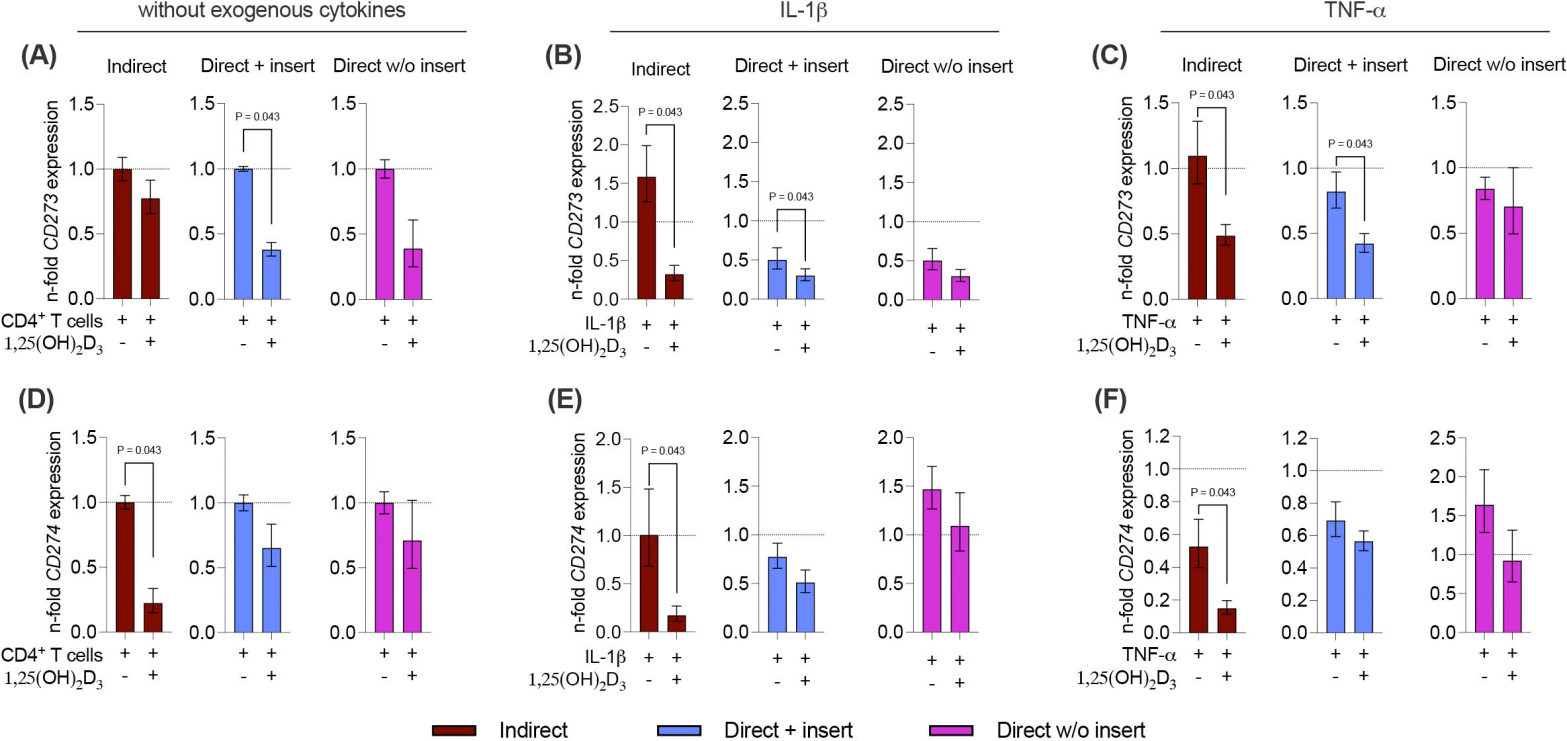
1,25(OH)_2_D_3_ partly reduces the gene expression of membrane-bound immunomediators in hPDL-MSCs independently from the co-culture models. After five days of co-cultivation, basal, IL-1β-, or TNF-α-triggered CD273 **(A-C)**, and CD274 **(D-F)** gene expression levels were determined in co-cultured hPDL-MSCs using qPCR. The y-axes show the n-fold expression compared to the appropriate controls within the different co-culture models. hPDL-MSCs co-cultured with CD4^+^ T lymphocytes in the absence of any cytokine and 1,25(OH)_2_D_3_ served as controls (n-fold expression = 1; shown by dotted lines). GAPDH was used as a reference gene. The data are presented as mean +/- standard error of the mean (S.E.M.). The data were obtained from five independent experiments with two technical replicates per experiment, using hPDL-MSCs from five distinct individuals. Statistically significant differences are shown by providing P-values obtained using the Wilcoxon Test for pairwise comparison.

Next, we also analyzed how 1,25(OH)_2_D_3_ influences the basal, IL-1β, or TNF-α-induced gene expression of the membrane-bound immunomediators in hPDL-MSCs in the different co-culture settings (**Figures 8A-F**). A decline in *CD273* and *CD274* gene expression levels by 1,25(OH)_2_D_3_ was observed, but statistically significant effects were only observed in some cases (**Figures 8A-F**). A significant decrease in *CD273* gene expression was detected in the direct co-culture model with insert, in the absence of exogenous cytokines (2.6-fold decrease; **Figure 8A**) and the presence of IL-1β (1.7-fold decrease; **Figure 8B**) or TNF-α (2-fold decrease; **Figure 8C**). Additionally, 1,25(OH)_2_D_3_ significantly reduced *CD273* gene expression in the indirect co-culture model in the presence of IL-1β (4.9-fold decrease; **Figure 8B**), and TNF-α (2.3-fold decrease; **Figure 8C**). *CD274* gene expression was significantly declined by 1,25(OH)_2_D_3_ in the indirect co-culture model, independently from the absence or presence of exogenous cytokines (**Figures 8D-F**). However, the extent of this suppression varies between a 4.5-fold, 5.9-fold, and 3.5-fold decline in the absence of exogenous cytokines (**Figure 8D**), and the presence of IL-1β (**Figure 8E**) and TNF-α (**Figure 8F**), respectively. Together, these data indicate that the effects of 1,25(OH)_2_D_3_ on *CD273* and *CD274* gene expression levels in hPDL-MSCs are partly suppressive depending on the co-culture models and the present exogenous cytokines.

## 4 Discussion

*In vitro* studies clearly showed the anti-inflammatory properties of 1,25(OH)_2_D_3_ against immune cells (25) and mesenchymal cells of the periodontium (27–29). In contrast, clinical studies investigating the relationship between vitamin D_3_ and various inflammatory diseases led to inconclusive results (33–36). This includes inflammatory bowel disease, obesity, and periodontitis, a chronic inflammation of the tooth-supporting tissue causing periodontal tissue destruction and tooth loss (37). Multiple studies demonstrated an inverse correlation between the 25-hydroxyvitamin-D_3_ serum level and the severity of periodontitis (38, 39), whereas another study observed an increased 25-hydroxyvitamin-D_3_ serum level with increased severity (40). Additionally, clinical studies only showed a moderate to no benefit of vitamin D_3_ supplementation after surgical and non-surgical periodontal treatment (41, 42). This inconsistency in the clinical and *in vitro* data may be due to the high complexity of the effects of vitamin D_3_ on periodontal tissues, which influences immune cells directly (25, 43) and indirectly via hPDL-MSCs (27) and even the reciprocal interaction between them (20, 44, 45). Our previous studies demonstrated the anti-inflammatory effects of 1,25(OH)_2_D_3_ on hPDL-MSCs by reducing IL-6, IL-8, and MCP-1 production (27) and on the bidirectional interaction between TNF-α- or IL-1β-treated hPDL-MSCs and CD4^+^ T lymphocytes (20). This study (20) only considered the paracrine interactions. However, data from our previously published study also demonstrated the contribution of direct cell-to-cell contact mechanisms to the reciprocal interaction between hPDL-MSCs and CD4^+^ T lymphocytes (22). Hence, investigating the effects of 1,25(OH)_2_D_3_ on the paracrine and direct cell-to-cell contact mechanisms will give us a more detailed picture.

The data of this study revealed that the effects of 1,25(OH)_2_D_3_ on CD4^+^ T lymphocyte proliferation and viability via hPDL-MSCs depend on the co-culture model, indicating that 1,25(OH)_2_D_3_ affects the paracrine and direct cell-to-cell contact mechanisms differently. Additionally, the degree of the impacts of 1,25(OH)_2_D_3_ on the cytokine production in CD4^+^ T lymphocytes differed depending on the distinct co-culture models. All these effects were verified by the 1,25(OH)_2_D_3_-based inhibition of immunomediator expression in hPDL-MSCs with different extents depending on the co-culture model.

The co-culture model greatly impacted the effects of 1,25(OH)_2_D_3_ on CD4^+^ T lymphocyte proliferation and viability via hPDL-MSCs. In the indirect and direct co-culture model with inserts, 1,25(OH)_2_D_3_ significantly inhibited the CD4^+^ T lymphocyte proliferation, which follows our previous study (20) and confirms its anti-inflammatory properties *in vitro*. This indirect anti-inflammatory effect seems to be executed via the paracrine signaling mechanisms between hPDL-MSCs and CD4^+^ T lymphocytes since this effect was exclusively observed in the indirect co-culture model. The direct co-culture conditions without inserts caused the opposite effect: increased CD4^+^ T lymphocyte proliferation in the presence of 1,25(OH)_2_D_3_-treated hPDL-MSCs. This suggests pro-inflammatory properties of 1,25(OH)_2_D_3_ against CD4^+^ T lymphocytes. It seems to be executed entirely via hPDL-MSCs, where the direct cell-to-cell contact mechanisms may play an essential role. This conclusion is made due to the following observations: First, in the absence of hPDL-MSCs, 1,25(OH)_2_D_3_ had a direct inhibitory effect on CD4^+^ T lymphocytes, as shown in our previous study under the same *in vitro* conditions (20). Second, this pro-inflammatory property can only be observed in the direct co-culture model without inserts, the only model allowing direct cell-to-cell contacts besides the paracrine interaction between hPDL-MSCs and CD4^+^ T lymphocytes (17–19). This effect of 1,25(OH)_2_D_3_ on CD4^+^ T lymphocyte proliferation in the direct co-culture model without inserts did not depend on the presence of the exogenous cytokines IL-1β and TNF-α.

The changes in CD4^+^ T lymphocyte proliferation induced by 1,25(OH)_2_D_3_-treated hPDL-MSCs inversely correlated with the alterations in the percentage of dead CD4^+^ T lymphocytes, independently from the used co-culture models. Hence, the inhibition of CD4^+^ T lymphocyte proliferation in the indirect and direct co-culture models with inserts is based on the induction of CD4^+^ lymphocyte death by 1,25(OH)_2_D_3_-treated hPDL-MSCs, which seems to be exclusively mediated by the paracrine interaction mechanisms between hPDL-MSCs and CD4^+^ T lymphocytes. In contrast, the enhanced CD4^+^ T lymphocyte proliferation in the direct co-culture model without inserts is predicated on reducing the very high number of dead CD4^+^ T lymphocytes caused by the direct cell-to-cell contacts with hPDL-MSCs.

Interestingly, the indirect and direct co-culture models with inserts led to identic results concerning the CD4^+^ T lymphocyte proliferation and viability. Additionally, these results were opposite to those obtained from the direct co-culture model without inserts. Hence, one can conclude that the direct co-culture model with inserts allows only minimal if any, direct cell-to-cell interactions between hPDL-MSCs and CD4^+^ T lymphocytes through the porous membrane.

The impact of the co-culture models on cytokine production by CD4^+^ T lymphocytes in the presence of hPDL-MSCs was less pronounced than that on proliferation and viability. The most predominant differences were observed between the direct co-culture without inserts and the two other models. Particularly, in the direct model without inserts, 1,25(OH)_2_D_3_ increased IL-10 production and did not affect the production of TNF-α, IL-22, and IL-5. In contrast, 1,25(OH)_2_D_3_ did not affect IL-10 production in the two other models and decreased TNF-α, IL-22, and IL-5 levels. A previous study (46) showed that IL-10 has an anti-apoptotic effect on T-lymphocytes; therefore, an anti-apoptotic effect of 1,25(OH)_2_D_3_ in a direct co-culture model without inserts could be related to increased IL-10 production. Furthermore, this anti-apoptotic effect could be partially associated with an increased CD4^+^ T lymphocyte proliferation by 1,25(OH)_2_D_3_ under these conditions. The production of some cytokines, particularly IFN-γ, IL-9, and IL-13, was inhibited by 1,25(OH)_2_D_3_ in all experimental models. Since specific CD4^+^ T lymphocyte subsets secrete these cytokines, the observed differences in cytokine secretion may indicate potential alterations in the CD4^+^ T lymphocyte subset composition. Not verifying the CD4^+^ T lymphocyte subset composition and their status after five days of co-cultivation represents a limitation of this study.

Compared to the co-culture models, the presence of the inflammatory cytokines IL-1β and TNF-α had only a limited impact on the effects of 1,25(OH)_2_D_3_ on proliferation, viability, and cytokine production by CD4^+^ T lymphocytes in co-culture with hPDL-MSCs. Exogenous IL-1β had almost no impact on the effects of 1,25(OH)_2_D_3_ on all parameters. The anti-apoptotic effect of 1,25(OH)_2_D_3_ on CD4^+^ T lymphocytes in a direct co-culture model without inserts was not observed in the presence of TNF-α. Similarly, the effect of 1,25(OH)_2_D_3_ on cytokine production by CD4^+^ T lymphocytes was qualitatively similar in the presence and in the absence of IL-1β. In contrast, some small alterations were detected in the presence of TNF-α. In particular, no stimulation of IL-10 production by 1,25(OH)_2_D_3_ in the direct co-culture model without inserts was observed. This finding agrees with the lack of an anti-apoptotic effect of 1,25(OH)_2_D_3_ under these conditions.

The reciprocal interaction between hPDL-MSCs and CD4^+^ T lymphocytes is versatile and includes soluble immunomediators and direct cell-to-cell contact mechanisms. Therefore, in the next step, we investigated the influence of 1,25(OH)_2_D_3_ on the expression of certain immunomediators in hPDL-MSCs under different co-culture settings. Our data predominantly showed a reduction of the soluble (*IDO-1, PTGS-2*, and *TSG-6*) immunomediators by 1,25(OH)_2_D_3_ in all co-culture models. However, some quantitative differences between the distinct models were observed. The most prominent differences were observed for *IDO-1*: the gene expression of *IDO-1* was inhibited by 1,25(OH)_2_D_3_ in both co-culture models with inserts without the presence of any exogenous cytokine and in the presence of IL-1β. However, this was not observed in the direct co-culture model without inserts under the same conditions. In the presence of TNF-α, 1,25(OH)_2_D_3_ decreased the *IDO-1* gene expression in all experimental models. The gene expression of *TSG-6* was decreased by 1,25(OH)_2_D_3_ in all co-culture models without any cytokine and in the presence of TNF-α, whereas no effect was detected in the presence of IL-1β. The gene expression of *PTGS-2* was decreased by 1,25(OH)_2_D_3_ under all experimental conditions. Since the effect of 1,25(OH)_2_D_3_ on the gene expression of the soluble immunomediators is predominantly suppressive, this cannot explain the differences in the hPDL-MSCs-based effects of 1,25(OH)_2_D_3_ on CD4^+^ T lymphocyte proliferation between the different co-culture models.

The gene expression of membrane-bound immunomediators (*CD273* and *CD274*) was predominantly diminished by 1,25(OH)_2_D_3_ at all conditions, but statistically significant differences were observed only in both co-culture models with inserts. In contrast, in the direct model without inserts, no significant effect of 1,25(OH)_2_D_3_ on the gene expression of both *CD273* and *CD274* was found. The PD-L1 protein, which is coded by the *CD274* gene, induces CD4^+^ T lymphocyte apoptosis with the PD-1 receptor on their surfaces (47), whereas PD-L2, encoded by the *CD273* gene, participates in inhibiting Th17 differentiation (48). Hence, it seems unlikely that the hPDL-MSCs-based anti-apoptotic effect of 1,25(OH)2D3 on CD4^+^ lymphocytes is associated with inhibiting *CD274* and *CD273* expression.

## 5 Conclusion

In conclusion, the hPDL-MSCs-based effects of 1,25(OH)_2_D_3_ on CD4^+^ T lymphocyte proliferation and viability strongly depend on the used co-culture setup, whereas the CD4^+^ T lymphocyte cytokine secretion seems to be influenced by a lesser extent. Since the three co-culture models vary in the reciprocal interaction between hPDL-MSCs and CD4^+^ T lymphocytes, it can be indicated that the distinct immunomodulatory mechanisms contribute differently to the observed plasticity of 1,25(OH)_2_D_3_. Besides the different co-culture setups, the data indicate that the local cytokine microenvironment is involved in fine-tuning the impact of 1,25(OH)_2_D_3_ on the reciprocal interaction between hPDL-MSCs and CD4^+^ T lymphocytes.

This study highlights the importance of using different co-culture models in parallel when investigating the immunomodulatory activities of hPDL-MSCs. Although the direct co-culture model without inserts is the best way to mimic the reciprocal interaction between hPDL-MSCs and immune cells *in vivo*, doing the indirect co-culture model in parallel can help distinguish between the involved paracrine and direct cell-to-cell contact mechanisms. Attention should also be given to which direct co-culture model is used since the comparability of the results between the indirect and direct co-culture model with inserts and the differences to the direct co-culture without insert indicate that a direct cell-to-cell interaction through the porous membrane in the direct co-culture with inserts is at least restricted or even completely impossible.

## Data Availability

The raw data supporting the conclusion of this article will be made available by the authors, without undue reservation.
